# Factors Associated with the Incidence of Local Recurrences of Breast Cancer in Women Who Underwent Conservative Surgery

**DOI:** 10.1155/2014/639534

**Published:** 2014-11-04

**Authors:** Juliana Rodrigues Tovar, Eliana Zandonade, Maria Helena Costa Amorim

**Affiliations:** ^1^Multivix Vitória College, 29043-900 Vitória, ES, Brazil; ^2^Program in Collective Health, Federal University of Espírito Santo, 29043-90 Vitória, ES, Brazil

## Abstract

Conservative surgery is considered the procedure of choice for women who are affected by early stage tumours. The local recurrence of cancer as a consequence of breast tissue conservation is a growing concern. This study aimed to describe the sociodemographic and clinical profiles of women who had local recurrences of breast cancer after conservative surgery and to examine the associations between sociodemographic and clinical variables and the incidence of tumour recurrence in these women. The retrospective cohort included 880 women who were diagnosed with breast cancer and underwent conservative surgery between January 2000 and December 2010. Recurrences occurred in 60 patients, and the mean age of the women at diagnosis was 48.8 years. Predictive factors for local recurrence were young age (<39 years) (*P* = 0.028 and OR = 10.93), surgical margin involvement (*P* = 0.001 and OR = 3.66), and Her-2 overexpression (*P* = 0.045 and OR = 1.94). The establishment of sociodemographic and clinical characteristics might help to select optimum treatments, which is a crucial challenge for public health in Brazil, especially with regard to reductions of surgery and hospitalisation expenditures in the Unified Health System (Sistema Único de Saúde—SUS).

## 1. Introduction

Worldwide, breast cancer is the most common type of cancer in women [1] and, in Brazil, the mortality rate from this disease increased between 1980 and 2006, thus making it a top priority noncommunicable chronic disease in the health care system [2].

In 2014, approximately 57,120 new cases of breast cancer in women were estimated by the National Cancer Institute (Instituto Nacional do Câncer (INCA)). Statistics for the State of Espírito Santo reported 990 cases in this primary location, and, in the city of Vitória (capital of the State of Espírito Santo), 130 new cases have been estimated [3].

Breast cancer treatment is currently initiated after an evaluation of lesion size, resection margin (whether affected or not), and the histopathological variety of the tumour. According to the Consensus Document for Control of Breast Cancer [4], the indications for different types of surgery depend on the clinical stage and histological type and can be conservative, which includes the removal of the tumour along with a surrounding surgical margin of healthy tissue (lumpectomy, extended resection, or quadrantectomy) and the axillary or sentinel lymph nodes and subsequent radiation, or nonconservative, which includes mastectomy, the total removal of the breast.

In the late nineteenth century, all women with breast cancer were submitted to radical mastectomy or* Halsted* surgery because little was known about the natural history of the disease [5]. Currently, breast-conserving surgery (BCS) is considered the procedure of choice for women who are affected by early stage tumours, and this procedure has been successful in terms of reducing invasive cancer therapy without compromising the results [6].

However, local cancer recurrence has become a major concern as a result of breast tissue preservation. The risk of recurrence has been a central theme of research in this area because failures in local control affect the survival of women and merit greater focus in plans for public policies that involve the health of women affected by breast cancer [7–10]. Age, tumour size, axillary lymph node involvement, surgical margins, histological type, hormone receptor expression, and systemic adjuvant treatment are factors that have been associated with the risk of local recurrence after BCS [11–15].

The objective of this study was to describe the sociodemographic and clinical profiles of women who suffered recurrences of breast cancer after BCS at Santa Rita de Cássia Hospital/Women's Association for Cancer Education and Control (Hospital Santa Rita de Cássia/Associação Feminina de Educação e Combate ao Câncer (HSRC/AFECC)) between 2000 and 2010 and to examine the associations between sociodemographic and clinical variables and the incidence of tumour recurrence in women who underwent conservative surgery.

## 2. Methodology

This was a retrospective cohort study performed by the examination of secondary data. The patient sample consisted of all women with breast cancer who underwent conservative surgery at HSRC-AFECC, which is located in Vitória, State of Espírito Santo, Brazil. The inclusion criteria were the existing case records of women with primary breast tumours who were registered at the institution during the period between 1 January 2000 and 31 December 2010 and who were initially treated with conservative surgery. The exclusion criterion was presentation with multiple primary tumours.

Research data were collected from the Cancer Hospital Registry (CHR), Sector of HSRC/AFECC, Philanthropic Hospital, and Oncology High Complexity Care Centre (Centro de Assistência de Alta Complexidade em Oncologia (CACON)) (ordinance MS number 741 of 19 December 2005) [16], which is located in Vitória, State of Espírito Santo, Brazil, during the period between 1 September 2011 and 31 May 2012. Prior to data collection, the researcher participated in training at the institution to acquire greater familiarity with the form-filling process.

The tumour registry forms and records represented the secondary data. A specific collection instrument was developed for forms that were not filled in or were incomplete. The assessed variables were as follows: age at diagnosis, race/ethnicity, primary tumour location, histological type, stage, laterality, tumour size, surgical resection margin involvement, number of axillary lymph nodes involved, sentinel lymph node biopsy technique, presence of vascular and/or lymphatic invasion, oestrogen and progesterone receptor expression, Her-2 and p53 tumour marker expression, local recurrence, and first treatment after surgery. Alcoholism, tobacco smoking, and family history of cancer variables were excluded from the analysis due to incompleteness of the data.

The data were organised with Microsoft Office Excel 2007 for Windows and were later processed with the Statistical Package for Social Sciences (SPSS) program, version 18.0.

A univariate descriptive analysis was performed on all data to ascertain the distribution patterns of women who were affected by breast cancer recurrences and treated at HSRC/AFECC. The results were analysed by calculating the frequencies, means, medians, and standard deviations. Two groups were formed according to the parameter of recurrence presence or absence and then were characterized as sociodemographic and clinical variables of the women with recurrent tumours. A chi-square independence test was applied to identify whether some of the sociodemographic and clinical characteristic variables were associated at a significance level of 5% (*P* < 0.05). Crude and logistic regression-adjusted odds ratios were subsequently calculated for the study variables to show statistical significance, and a significance level of 0.10 was adopted for this purpose.

This study was submitted to and approved by the Centre for Education and Research of HSRC/AFECC and the Research Ethics Committee of the Health Sciences Centre (Comitê de Ética em Pesquisa do Centro de Ciências da Saúde) of the Federal University of Espírito Santo in accordance with resolution number 196 of October16, 1996 [17] and research project number 240/11.

## 3. Results

Initially, 1,759 patients with breast tumours for whom surgery was the initial treatment were registered during the period from January 1, 2000 to December 31, 2010. Eight men and 815 women were excluded for whom radical mastectomy was the initial treatment, along with 19 women for whom the initial treatment was quadrantectomy followed by radical mastectomy (performed due to involvement of the initial surgery margins as detected by histopathology). However, 22 cases of bilateral tumours remained, of which 11 were excluded after drawing lots to determine the laterality (right or left) that would remain in the study for data analysis. Twenty-six women who did not remain in the institution for monitoring for more than 2 months were also excluded. Ultimately, 880 women with breast cancer for whom quadrantectomy was the initial treatment performed at the institution were included in the study.

Recurrences occurred in 60 (6.8%) of these women, and the mean age at diagnosis was 48.8 years (median: 49 years, standard deviation: 12.2). The minimum and maximum ages were 30 and 101 years old, respectively.

To describe the characteristics of women who had local recurrence of cancer, Table 1 presents the sociodemographic and clinical variables of these patients.

The majority of women were aged from 30 to 49 years (57%), and most were nonwhite (77%). Among the clinical characteristics, 58% of the primary tumours were located in the upper outer quadrant, and the most prominent histological types were ductal and mixed carcinoma (90%). The vast majority of cases (93%) involved an initial-stage tumour. Twenty-nine (49%) women had tumours with diameters larger than 2 cm, 45 (76%) had no vascular invasion, and 48 (80%) had negative surgical margins. There was no axillary lymph node involvement in 70% (39) of the women with recurrences, and the sentinel nodes were identified by vital blue dye in 88% (23) of the cases. Most of the tumours were hormone receptor positive, whereas p53 and Her-2 expression were negative in much of the sample.

In the elapsed time (in months) between conservative surgery and the local recurrence of breast cancer, a mean time of 35.5 months (median: 28.5, standard deviation: 27.5) was observed, with minimum and maximum times of 2 and 112 months, respectively.

Table 2 shows the association between the sociodemographic and clinical variables and the rate of recurrence in women with breast cancer who underwent conservative surgery. The variables race/ethnicity, location of primary tumour, histological type, stage, laterality, vascular invasion, axillary lymph node involvement, sentinel lymph node biopsy, estrogen receptor (RE), progesterone receptor (PR), and p53 were not statistically significant at the 5% level. Significant variables associated with recurrence were less than 39 years (*P* = 0.003), chemotherapy as the initial treatment after surgery (*P* = 0.028), tumour size greater than 2 cm (*P* = 0.006), positive involvement of surgical margins (*P* = 0.001), and Her-2 expression (*P* = 0.016).

Table 3 shows the crude* odds ratio* and the odds ratio adjusted by the logistic regression model of the statistically significant variables (0.10 level of significance). After adjustment, statistical significance was only retained for the following variables: less than 39 years (*P* = 0.028 and OR = 10.93), surgical margin involvement (*P* = 0.001 and OR = 3.66), and Her-2 expression (*P* = 0.045 and OR = 1.94).

## 4. Discussion

This study took place at HSRC-AFECC, the reference cancer treatment hospital in the State of Espírito Santo. This hospital also receives demand from residents of states that border ES, making it a statewide representative hospital in the service of populations affected by breast cancer. One purpose of the information collected from the institutional CHR is to monitor registered cancer cases, thus allowing professionals to access statistical data on the outcomes of proposed treatments [18]_._

In total, 880 medical records of women with breast cancer who had undergone BCS as the initial treatment were reviewed. From an analysis of the characteristics of women who had local recurrences of primary tumours, a profile was identified for these women, along with certain factors that were associated with recurrence.

The local recurrence rate of 6.8% was lower than the rate of 7.8% that was reported in another Brazilian study [14]. Data from the literature show that 4–20% of women with BCS-treated breast cancer experience tumour recurrences [7, 12, 19].

The mean time to recurrence of 35.53 months is similar to that reported in a Japanese study, which observed that the majority of recurrences (60.2%) occurred during the first 30 months after surgery [20], and in a Brazilian study [1, 4], which reported a time of 31.4 months for a group of patients with true local recurrences. American guidelines for women with breast cancer recommend a follow-up period of 3 to 5 years. However, other important researches have revealed that the risk pattern of locoregional recurrence remains for up to 10 years [21], which corroborates our findings.

Most women were in the 30- to 49-year age group at the time of diagnosis, and the mean age was 48.8 years. A very similar result was reported in a Spanish study, wherein the mean age was 50 years; however, the survival rate was lower in young premenopausal women [22]. In Brazil, the Ministry of Health recommends an annual clinical breast examination for women over 40 years of age and a biannual mammography for women from 50 to 69 years for early detection and diagnosis [4]. However, there is no specific indication that women aged between 40 and 49 years should undergo mammography (screening or diagnostic), although this is recommended for women of 35 years and older who have a family history of the disease. However, the law 11.664/2008 has guaranteed access to mammography for women aged 40 years and older and has thus become a legal landmark in the fight against breast cancer [23].

The impact of race on disease prognosis is not fully understood with regard to women treated with BCS. A recent retrospective analysis of 1,231 women aged 40 years or more with initial-stage invasive tumours who were treated with conservative surgery and radiotherapy at the University of Chicago Hospital between 1986 and 2004 showed that black women had a worse overall survival rate (64.6%) than nonblack women (80.8%) [24]. The predominance of nonwhite patients in our study only reflects the race/ethnicity ratio in the general population of the state of ES [25] and does not allow us to draw any further associations. However, the social inequalities in Brazil have been widely described and are related to health service access; thus, this finding merits attention, as it demonstrates that race relations are an issue that should guide the discussion on citizenship in the country [26].

Regarding primary tumour location, the upper outer quadrant is the most frequent [27], which is similar to observations in the present study. Additionally, the left breast was more commonly affected in other studies [27, 28]. This was not observed in the present study, where the majority of tumours were found in the right breast, although the difference between the two lateralities was low (47% cases in the left breast and 53% in the right). The lack of studies that relate this variable to profiles of breast cancer recurrence in women who underwent conservative surgery precludes us from making more specific comparisons. The tumour location in these cases is considered when comparing the recurrent tumour to the primary tumour, which did not occur in the present study. The results from a Japanese study showed that 79% of recurrence cases occurred in the same quadrant, as opposed to 49% of cases of new primary tumours, indicating a larger number of cases of new tumours (51%) that occur in other quadrants [29].

Invasive ductal carcinoma was the most frequent histological type. According to the literature, this histological type generally represents between 70 and 80% of cases [27]. In a similar cohort in Mexico, 23% (the majority) of local recurrence cases were reported to be invasive and ductal [13]. In Brazil, a study conducted in São Paulo [30] found that 83.1% of the cases presented with the same histological type, thus corroborating the profile of women with breast cancer who were treated at the same hospital as those in the present study (HSRC/AFECC) [31]; the prevalence in the latter study was 86%.

We found that the majority of women were diagnosed at an early stage, which was a prerequisite for conservative surgery in accordance with the Consensus [4] recommendations.

Vascular and/or lymphatic invasion was absent in most women, and other authors [11, 32] who have studied the profiles of women with breast cancer who underwent BCS reported similar findings. However, other authors reported lower survival rates for women whose tumours had vascular invasion [32], as well as associations with metastasis and death after BCS [11].

The lack of lymph node involvement corroborates an Italian study demonstrating that, of 32 women whose tumours recurred within 5 years of conservative surgery, 17 had no lymph node involvement [11]. In a Canadian prospective cohort study of 1,540 women with negative lymph nodes who underwent conservative surgery, a local recurrence rate of 6.4% and a mortality rate of 7.6% [15] were found.

With regard to sentinel lymph node (SLN) detection, a Brazilian study evaluated the effectiveness of the various existing methods and demonstrated that both the use of technetium, applied between 3 and 17 hours before surgery, and the blue dye are acceptable methods for SLN mapping. However, the efficiency was 100% when both methods were used [33].

Only 21% of women were positive for p53 expression, while 79% were negative. Studies have shown that increased p53 expression is associated with increased tumour aggressiveness and a worse prognosis [28].

The predominance of hormone receptor positivity in this study was corroborated by the reviewed literature, in which approximately two-thirds of breast carcinomas were reported to be positive for progesterone (PR) and estrogen receptor (ER) expression. This expression was strongly associated with age and was inversely proportional to tumour size and histological and nuclear grades. However, hormone receptor positivity represents a better prognosis and a more satisfactory response to hormone therapy [28].

In the reviewed literature [34], the authors described an increased risk of local recurrence in younger women who underwent conservative surgery. Proposed hypotheses to explain this fact have been based on pathological characteristics, including a higher prevalence of extensive intraductal components, a greater prevalence of higher nuclear grades, more aggressive biological behaviour, or an increased likelihood of residual disease after the initial biopsy. Lower responses to chemotherapy or low doses of RT have been suggested.

The tumour size and the affected surgical margins corroborate the findings of other studies [11, 15, 32, 34]. According to the Consensus document for the Control of Breast cancer that was established by INCA [4], a tumour larger than 2 cm has characteristics that indicate a high risk of recurrence. A study of 50 women with positive margins at the initial excision showed that 21 women (42%) had residual tumour in the second excision, thus confirming this recommendation. More specifically, 88% of women with tumours larger than 2 cm in size and 93% of those with extensive margin involvement (≥1.0 cm) had positive results in repeated excisions, indicating that tumour size and surgical margin involvement correlate with a higher probability of presenting with a residual lesion [34].

Moreover, tumour size* per se* is not an exclusion criterion for conservative surgery unless it would result in a poor aesthetic appearance due to small breast size. Two major prospective studies showed no significant differences in the survival rates of women with tumours larger than 4 cm who underwent conservative surgery [6].

Young patient age has been reported to be a poor prognostic factor among women with local recurrence, but it is not clear which factors contribute to this [35, 36]. In contrast to the present study, another one found that early age of diagnosis was a good prognostic factor. Although young patients experience more LR than older ones, once LR occurs, young patients had a better outcome than the others [37].

An Italian study [11] also described an association between local recurrence and Her-2 positivity, wherein this variable was predictive of regional recurrences and distant metastases or death. Her-2 is a protooncogene that occurs in approximately 20% of all breast cancers and suggests a poor prognosis, although most positive tumours respond better to nonhormonal chemotherapy [28]. Particularly in pT1AbN0M0 breast cancers, Her-2 overexpression was correlated with a risk factor for recurrence [38, 39] and death [39] and was also a significant risk factor for 1-year disease-free survival and distant disease-free survival [40].

The standard of care for adjuvant therapy of early breast carcinoma includes whole breast irradiation in case a breast-conserving strategy is applicable. The purpose of the irradiation is to minimise the risk of local failure and the strategy includes irradiating the mammary gland and in node-positive patients also locoregional lymph nodes with doses around 50 Gray in 25 fractions delivered as daily treatment 5 days per week for 5-6 weeks [41].

However, many studies have shown that hypofractionated radiotherapy has been a safe and effective option for patients with breast cancer in early stage; it provides a lower total radiation dose given in a few slightly larger fractions and delivered over a short period time by use of hypofractionation schedules [42].

According to a study published by the American Society of Clinical Oncology, the best way to integrate chemotherapy and radiotherapy after BCS for women with breast cancer remains unknown. Based on this statement, the authors conducted a randomised trial that demonstrated a higher risk of local recurrence in women who received chemotherapy before radiotherapy after surgery [38]. Although not statistically significant, that study agrees with our findings; specifically that chemotherapy as the initial treatment after surgery was statistically associated with local recurrence.

However, a limitation must be considered. Namely, the quality of the results might have been affected by the incompleteness of some variables, which were caused by mistakes in record completion.

## 5. Conclusions

This is a pioneering study for the State of Espírito Santo. Out of a sample of 880 women who underwent conservative surgery, 60 (6.8%) had local recurrences of breast cancer, and the predictive factors for the incidence of this event were young age, surgical margin, and positive expression of the Her-2 tumour marker.

As the research was conducted at a single institution in Vitória, other studies should be conducted to ascertain the characteristics of women who are in the “at risk” group to support efficient policies for the early detection of breast cancer and to significantly change this profile.

The lack of data in the records reflects the lack of real dimension possessed by health professionals with regard to this information. Improved data quality from these professionals is of extreme importance for the implementation of public policies and for the performance of epidemiological studies. Surveys of secondary data are extremely important to understand the reality of the area and to plan new clinical strategies and policies that are aimed at the better targeting and treatment of women.

The definition of risk for the development of local recurrence in women is fundamental when deciding the eligibility of each patient for the most appropriate protocol. The establishment of sociodemographic and clinical characteristics might help in the selection of the best treatments, which is a crucial challenge for public health, especially with regard to the reductions in surgery and hospitalisation SUS spending, increased patient quality of life and survival of patients, and reductions in the psychological effects that cancer recurrences present to these women.

## Figures and Tables

**Table 1:** Sociodemographic and clinical characterisation of women with recurrent breast cancer who underwent breast-conserving surgery and were enrolled in the HSRC during the period from January 1, 2000 to December 31, 2010.

Variable	Category	*N* = 60	%
Age group	30 to 39 years	**13**	**22%**
	40 to 49 years	**21**	**35%**
	50 to 59 years	16	27%
	60 to 69 years	9	15%
	80 or more	1	2%

Race/ethnicity	White	14	23%
	Nonwhite	**46**	**77%**

Location of primary tumour	Nipple	2	5%
	Upper inner quadrant	8	21%
	Lower inner quadrant	5	13%
	Upper outer quadrant	**22**	**58%**
	Lower outer quadrant	1	3%

Histological type	Others	1	2%
	Ductal and mixed carcinoma	**54**	**90%**
	Lobular carcinoma	5	8%

Stage	Initial	**56**	**93%**
	Late	4	7%

First postsurgery treatment	RT	22	36.7%
	CT	**36**	**60.0%**
	Hormone	2	3.3%
	None	0	0%

Laterality	Right	**32**	**53%**
	Left	28	47%

Size in cm	0.5 or less	3	5%
	0.6 to 1.0	6	10%
	1.1 to 1.5	5	8%
	1.6 to 2.0	16	27%
	>2.0	**29**	**49%**

Vascular and/or lymph invasion	No	**45**	**76%**
	Yes	14	24%

Surgical margin involvement	Negative	**48**	**80%**
	Positive	12	20%

Number of axillary lymph nodes involved	0	**39**	**70%**
	1–3	12	21%
	>3	5	9%

Sentinel lymph node detection technique	Dye	**23**	**88%**
	Radiopharmaceutical	2	8%
	Both	1	4%

Estrogen receptor	Negative	17	29%
	Positive	**41**	**71%**

Progesterone receptor	Negative	18	31%
	Positive	**40**	**69%**

Her-2	Negative	**35**	**65%**
	Positive	19	35%

p53	Negative	**31**	**69%**
	Positive	14	31%

RT: radiotherapy, CT: chemotherapy.

**Table 2:** Relationships between sociodemographic and clinical variables and the incidence of breast cancer recurrence in women who underwent breast-conserving surgery and were registered in HSRC during the period from January 1, 2000 to December 31, 2010.

Variable	Category	Recurrence				*P* value
		No		Yes		
		*N* = 820	%	*N* = 60	%	
Age group	Less than 39 years	**76**	**9%**	**13**	**22%**	**0.003**
	40 to 49 years	230	28%	21	35%	
	50 to 59 years	240	29%	16	27%	
	60 to 69 years	174	21%	9	15%	
	70 years or more	100	12%	1	2%	

Race/ethnicity	White	278	34%	14	23%	0.086
	Nonwhite	536	66%	46	77%	

Location of primary tumour	Nipple	22	4%	2	5%	0.539
	Upper inner quadrant	61	12%	8	21%	
	Lower inner quadrant	56	11%	5	13%	
	Upper outer quadrant	324	66%	22	58%	
	Lower outer quadrant	28	6%	1	3%	

Histological type	Ductal and mixed carcinoma	703	86%	54	90%	0.075
	Lobular carcinoma	40	5%	5	8%	
	Others	77	9%	1	2%	

Stage	Initial	611	95%	56	93%	0.570
	Late	32	5%	4	7%	

First postsurgery treatment	None	42	5%	0	0%	**0.028**
	RT	388	47%	22	37%	
	CT	**345**	**42%**	**36**	**60%**	
	Hormone	45	5%	2	3%	

Laterality	Right	408	51%	32	53%	0.678
	Left	399	49%	28	47%	

Size in cm	0.5 or less	60	8%	3	5%	**0.006**
	0.6 to 1.0	149	19%	6	10%	
	1.1 to 1.5	181	23%	5	8%	
	1.6 to 2.0	152	19%	16	27%	
	>2.0	**257**	**32%**	**29**	**49%**	

Vascular and/or lymph invasion	No	678	85%	45	76%	0.095
	Yes	124	15%	14	24%	

Surgical margin involvement	Negative	752	93%	48	80%	**0.001**
	Positive	**54**	**7%**	**12**	**20%**	

Lymph node involvement	0	555	75%	39	70%	0.672
	1–3	126	17%	12	21%	
	>3	63	8%	5	9%	

Sentinel lymph node detection technique	Dye	433	90%	23	88%	0.913
	Radiopharmaceutical	28	6%	2	8%	
	Both	22	5%	1	4%	

Oestrogen receptor	Negative	141	20%	17	29%	0.088
	Positive	568	80%	41	71%	

Progesterone receptor	Negative	173	25%	18	31%	0.270
	Positive	533	75%	40	69%	

Her-2	Negative	517	79%	35	65%	**0.016**
	Positive	**138**	**21%**	**19**	**35%**	

p53	Negative	345	79%	31	69%	0.129
	Positive	93	21%	14	31%	

RT: radiotherapy, CT: chemotherapy.

**Table 3:** *Crude and adjusted odds ratios* for statistically significant variables in women with breast cancer who underwent breast-conserving surgery and who were registered in HSRC during the period from January 1, 2000 to December 31, 2010.

Variables	Categories	Crude OR				Adjusted OR^*^			
		*P* value	OR	LI 95%	LS 95%	*P* value	OR	LI 95%	LS 95%
Age group	Less than 39 years	**0.007**	**17.11**	**2.19**	**133.63**	**0.028**	**10.93**	**1.29**	**92.66**
	40 to 49	0.032	9.13	1.21	68.81	0.083	6.28	0.78	50.23
	50 to 59	0.068	6.67	0.87	50.95	0.118	5.33	0.65	43.49
	60 to 69	0.122	5.17	0.65	41.43	0.147	4.89	0.57	41.86
	70 years and over		1.00				1.00		

Race/ethnicity	White		1.00				1.00		
	Nonwhite	0.090	1.70	0.92	3.15	0.488	1.29	0.63	2.64

Histological type	Ductal and mixed carcinoma	0.080	5.91	0.81	43.35	0.997	1.50	0.10	4.00
	Lobular carcinoma	0.042	9.62	1.09	85.21	0.997	1.30	0.20	3.50
	Others		1.00				1.00		

First treatment after surgery	None	0.998	∗∗	∗∗	∗∗	0.998	∗∗	∗∗	∗∗
	RT	0.747	1.28	0.29	5.60	0.886	0.89	0.18	4.40
	CT	0.251	2.35	0.55	10.08	0.789	0.80	0.16	4.05
	Hormone		1.00				1.00		

Size in cm	≤0.5		1.00				1.00		
	0.6–1.0	0.765	0.81	0.20	3.33	0.413	0.52	0.11	2.47
	1.1–1.5	0.426	0.55	0.13	2.38	0.257	0.41	0.09	1.93
	1.6–2.0	0.250	2.11	0.59	7.49	0.866	1.13	0.27	4.67
	>2.0	0.192	2.26	0.67	7.66	0.673	1.34	0.34	5.27

Vascular and/or lymphatic invasion	No		1.00				1.00		
	Yes	0.098	1.70	0.91	3.19	0.606	1.22	0.58	2.57

Surgical margin involvement	Negative		1.00				1.00		
	Positive	**0.000**	**3.48**	**1.75**	**6.94**	**0.001**	**3.66**	**1.65**	**8.09**

Oestrogen receptor	Negative		1.00				1.00		
	Positive	0.091	0.60	0.33	1.09	0.101	0.56	0.29	1.12

Her-2	Negative		1.00				1.00		
	Positive	**0.018**	**2.03**	**1.13**	**3.67**	**0.045**	**1.94**	**1.02**	**3.72**

^*^The total data analysed were based on 676 women (76.8%), due to missing data in at least one variable.

^**^could not be calculated because no women with local recurrences remained in this analysis category.

RT: radiotherapy, CT: chemotherapy.
